# Dosing of anti-infective drugs in patients undergoing therapeutic plasma exchange – considerations for practical application: a narrative review

**DOI:** 10.1186/s13054-026-06306-0

**Published:** 2026-09-24

**Authors:** Maj-Britt Nachtigall, J. J. Schmidt, S. Scherneck, J. Flechtner, S. David, J. T. Kielstein

**Affiliations:** 1 Nephrology | Rheumatology | Blood Purification, Academic Teaching Hospital Braunschweig, Braunschweig, Germany; 2 Institute of Pharmacology, Toxicology and Clinical Pharmacy, Technische Universität Braunschweig, Mendelssohnstrasse 1, 38106 Braunschweig, Germany; 3 Department of Nephrology and Hypertension, Hannover Medical School, Hannover, German Germany; 4 University Department of Internal Medicine, Section Nephrology and Hypertension, Klinikum Oldenburg AöR, Oldenburg, Germany; 5 Institute of Intensive Care Medicine, University Hospital Zurich & University of Zurich, Zurich, Switzerland

**Keywords:** Therapeutic plasma exchange, Plasmapheresis, Pharmacokinetics, Anti-infectives

## Abstract

**Background:**

Therapeutic plasma exchange (TPE) is used in a wide range of indications and patient populations. The current guidelines of the American Society for Apheresis on the use of therapeutic apheresis in clinical practice list 166 graded indications for TPE. Many indications are related to the treatment of critically ill patients. Indeed, two multicenter trials are currently investigating the role of TPE in the treatment of early septic shock.

**Main body:**

During TPE, usually 1.0–1.5 times the calculated plasma volume is exchanged using albumin solution or fresh frozen plasma as replacement fluid, although considerable deviations from this recommended exchange volume have been reported. Depending on the TPE modality (membrane-based or centrifugal), the duration of the procedure ranges between 90 and 140 min. TPE removes not only unwanted substances such as inflammatory mediators or pathological antibodies but may also remove drugs that are essential for the treatment of the underlying disease. Furthermore, changes in plasma protein concentrations due to TPE may affect the volume of distribution (Vd) and the concentrations of protein-bound drugs. The objective of the current narrative review is to introduce general principles of TPE-associated drug removal as well as the current literature on the dosing of anti-infectives in patients undergoing TPE.

**Conclusion:**

Pharmacokinetic characteristics, including volume of distribution, plasma protein binding, endogenous clearance, and redistribution kinetics, have been demonstrated to influence the susceptibility of a drug to TPE-associated removal. TPE may substantially affect plasma levels of drugs with low volume of distribution and high protein binding, including aminoglycosides and glycopeptides. Further studies are needed because direct evidence on the pharmacokinetics of anti-infective drugs undergoing TPE in sepsis remains limited. Specific timing or dosing strategies are therefore not supported by sufficient evidence and should be individualized according to drug-specific pharmacokinetics, TPE characteristics, and clinical situation.

**Graphical Abstract:**

**Figure:**
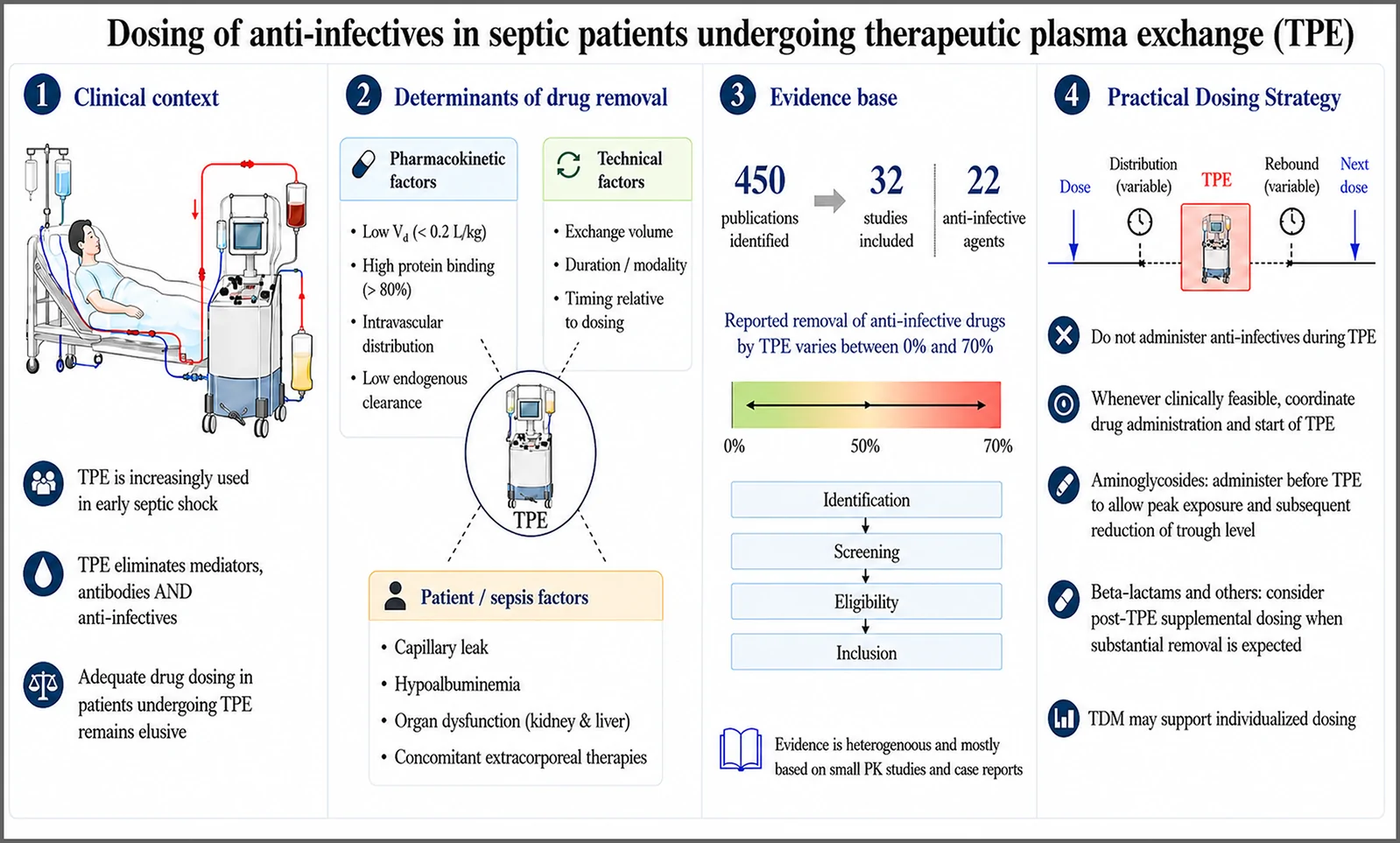

**Supplementary Information:**

The online version contains supplementary material available at https://doi.org/10.1186/s13054-026-06306-0.

## Key message

Most anti-infectives are not significantly removed by TPE.

TPE may influence drug levels of anti-infectives to a significant extent, during the early distribution phase, so drugs should not be given during the TPE.

The interval between initial anti-infective administration and TPE initiation varies across clinical settings and should not be assumed to prevent clinically relevant TPE-associated drug removal.

The theoretical maximum TPE-associated clearance is limited by the plasma removal rate, whereas total drug removal undergoing a TPE session additionally depends on treatment duration and plasma drug concentration.

When aminoglycoside administration and TPE are scheduled in close temporal proximity, administration before TPE may be considered if sufficient time is available to achieve adequate peak exposure and initial distribution. For other anti-infectives, dosing relative to TPE should be individualized according to drug-specific pharmacokinetics, expected TPE-associated drug removal, and clinical situation.

**Supplementary Information:**

The online version contains supplementary material available at https://doi.org/10.1186/s13054-026-06306-0.

## Introduction

There is growing research interest in TPE as an adjunctive treatment for severe immune-mediated and inflammatory conditions in critically ill patients, including sepsis-associated syndromes [1]. After small early trials in the 1990s and 2000s suggested a possible survival benefit and reversal of multi-organ dysfunction syndrome in sepsis with TPE [2], small prospective trials conducted in the 2020s supported these findings [3], which were subsequently reinforced by meta-analyses [4, 5]. While the results of adequately powered prospective trials of TPE in early sepsis, such as the EXCHANGE-2 trial [6], are awaited, the question of how to ensure timely and adequate antimicrobial therapy in septic patients undergoing TPE remains unresolved. This pharmacological treatment remains the cornerstone of sepsis management, with early attainment of pharmacokinetic/pharmacodynamic (PK/PD) targets strongly associated with improved outcomes [2]. However, the concomitant use of TPE and anti-infective therapy introduces significant and often underappreciated challenges in drug dosing. TPE has the potential to substantially alter the pharmacokinetics of anti-infective agents through extracorporeal drug removal, particularly for compounds with low volume of distribution, high plasma protein binding, or limited tissue penetration. The extent of TPE-associated drug removal is influenced by multiple factors, including the TPE modality (membrane-based versus centrifugal), exchanged plasma volume relative to the patient’s calculated plasma volume, replacement-fluid composition, and the timing of antimicrobial administration relative to the procedure. In septic patients, these complexities are further compounded by pathophysiological alterations such as capillary leak, hypoalbuminemia, augmented renal clearance, or concomitant renal replacement therapy (RRT), all of which contribute to high inter- and intra-individual variability in drug exposure. Despite the clinical relevance of these interactions, dosing recommendations for antibiotics during TPE remain poorly standardized and are often extrapolated from limited pharmacokinetic data, case reports, or small cohort studies. This uncertainty poses a risk of subtherapeutic exposure, treatment failure, and the emergence of antimicrobial resistance, particularly in the context of severe infections requiring optimal and sustained drug concentrations.

In this review, we summarize the current evidence on the impact of TPE on anti-infective pharmacokinetics, identify key drug- and procedure-related determinants of TPE-associated drug removal, and discuss practical considerations for dose adjustment and therapeutic drug monitoring. Our aim is to provide clinicians with a structured framework to optimize antimicrobial therapy in septic patients undergoing TPE, while highlighting critical knowledge gaps and priorities for future research.

## Background

### Factors influencing drug removal undergoing TPE

#### Volume of distribution (V_d_)

The V_d_ represents a theoretical volume that would be necessary to hold the absolute amount of a given drug at the plasma concentration after distribution ($\:{V}_{d}=\:\frac{Drug\:amount}{Plasma\:concentration}$). Therefore, the V_d_ describes the extent to which a drug distributes into different body compartments and thereby reflects the theoretical maximum proportion of drug that can be removed by extracorporeal therapies with access limited to the plasma compartment. TPE is assumed to decrease the plasma concentration of a solely intravascular substance during the first treatment session by 70–75%. This is followed by a decrease of 23% during the second and 9% during the third session. However, the impact of single or repeated TPE treatments on drug concentrations depends largely on a drug’s V_d_ and its redistribution kinetics between the extra- and intravascular compartments [3]. 

#### Plasma protein binding (PPB)

The PPB describes the fraction of a drug in plasma that is bound to proteins. The unbound fraction is generally pharmacologically active, whereas the protein-bound fraction may act as a reservoir and is usually less readily cleared or metabolized. However, TPE may also remove the protein-bound drug fraction and thereby contribute to total drug clearance in highly protein-bound drugs. Therefore, a low V_d_ (< 0.2 L/kg) and a high PPB (> 80%) are associated with increased TPE-associated drug removal [4]. 

#### Lipophilicity and degree of ionization

The V_d_ is influenced by the physicochemical properties of a drug, particularly lipophilicity and the degree of ionization. Lipophilic drugs can pass the phospholipid membrane and accumulate easily in body tissues, resulting in a larger V_d_. Conversely, hydrophilic drugs remain in the bloodstream or extracellular fluid, resulting in a smaller V_d_. The degree of ionization also impacts the V_d_: Non-ionized molecules pass through the phospholipid membrane more easily than ionized ones. Therefore, a drug that is lipophilic and poorly ionized will distribute extensively throughout the body and have a high V_d_. Conversely, a hydrophilic and highly ionized drug will have a low V_d_ and remain primarily in the vascular compartment. In general, hydrophilic, ionized drugs are more affected by TPE because a higher drug concentration is available in the plasma. This is especially true when the PPB is high [5]. 

While physicochemical properties such as lipophilicity, ionization, and protein binding allow for a rough estimation of a drug’s volume of distribution, these considerations are typically based on simplified one-compartment models. In real life, however, many drugs follow more complex distribution patterns. Certain anti-infectives, including vancomycin and gentamicin, distribute according to multi-compartmental kinetics, meaning they do not spread evenly throughout the body, but rather bind with varying affinities to different tissue and fluid compartments over time. This behavior can significantly influence drug levels, especially during interventions like TPE, where redistribution between compartments may not occur immediately. As a result, predicting the extent of TPE-associated drug removal becomes more challenging, and general assumptions based on physicochemical properties alone may be insufficient. Therefore, drug-specific studies in critically ill patients are essential to establish reliable dosing strategies during TPE [6]. 

#### Exchanged plasma volume

For most indications the current ASFA guidelines recommend an exchange volume of 1.0–1.5 times of the calculated plasma volume, which can be calculated by different equations (e.g. Nadler’s Eq. [7]). Real-world data suggest that the intended TPE intensity is not consistently delivered. In a single-center study from a German tertiary-care hospital, 45% of almost 1,000 TPE procedures were performed with an exchange volume < 1.0 times the calculated plasma volume [13]. Economic constraints, i.e. fixed reimbursement models that do not adequately account for the costs of blood products, may contribute to the use of relatively fixed exchange volumes in routine clinical practice [8]. Consequently, even tertiary care centers may perform TPE with exchange volumes ranging from only 0.4 to 1.0 times the calculated plasma volume [9]. A study from India reports an overall exchange volume as low as 2097 ± 916 ml [10]. At the other end of the TPE dose spectrum, exchange volumes of 60 mL/kg actual body weight were used in the PEXIVAS trial [17], while high-volume plasma exchange of up to 12 L has been reported in acute liver failure [18]. 

#### Treatment duration

Treatment duration and exchanged plasma volume are important determinants of the TPE-associated drug removal, as both factors directly influence the exposure of circulating drugs to extracorporeal removal. In a single-center study investigating 912 TPE treatments, treatment duration was shorter with centrifugal techniques (120.0 [112.5–135.0] min) than with membrane-based TPE (135.0 [125.0–140.0] min) [13]. Depending on the applied technique and treatment setting, procedure duration may be as short as 100 min [11]. 

#### Distribution and redistribution kinetics

The extent of TPE-associated drug removal depends on the fraction of the drug present in the intravascular compartment at the time of the procedure. If an anti-infective is administered immediately before or during TPE, a larger fraction may still be accessible to extracorporeal removal before distribution into extravascular compartments has occurred [12]. For drugs exhibiting multi-compartment pharmacokinetics, distribution may comprise several phases with different rates, and a single distribution half-life may therefore not adequately describe this process. Redistribution from extravascular compartments may further influence plasma concentrations during and after TPE [13]. Consequently, the timing of TPE relative to drug administration should be considered in relation to drug-specific distribution and redistribution kinetics rather than distribution half-life alone.

#### Extracorporeal clearance undergoing TPE

In general, the maximum possible clearance of a drug during TPE corresponds to the plasma removed over time. In the example of meropenem, which has a plasma clearance of approximately 15.5 L/h in a 70 kg person, the exchange of 4000 ml plasma over two hours would increase total plasma clearance from 15.5 to 17.5 L/h during the procedure. Thus, for drugs with already high endogenous clearance, the additional extracorporeal clearance provided by TPE may be clinically negligible, particularly when treatment duration is short.

**Fig. 1:**
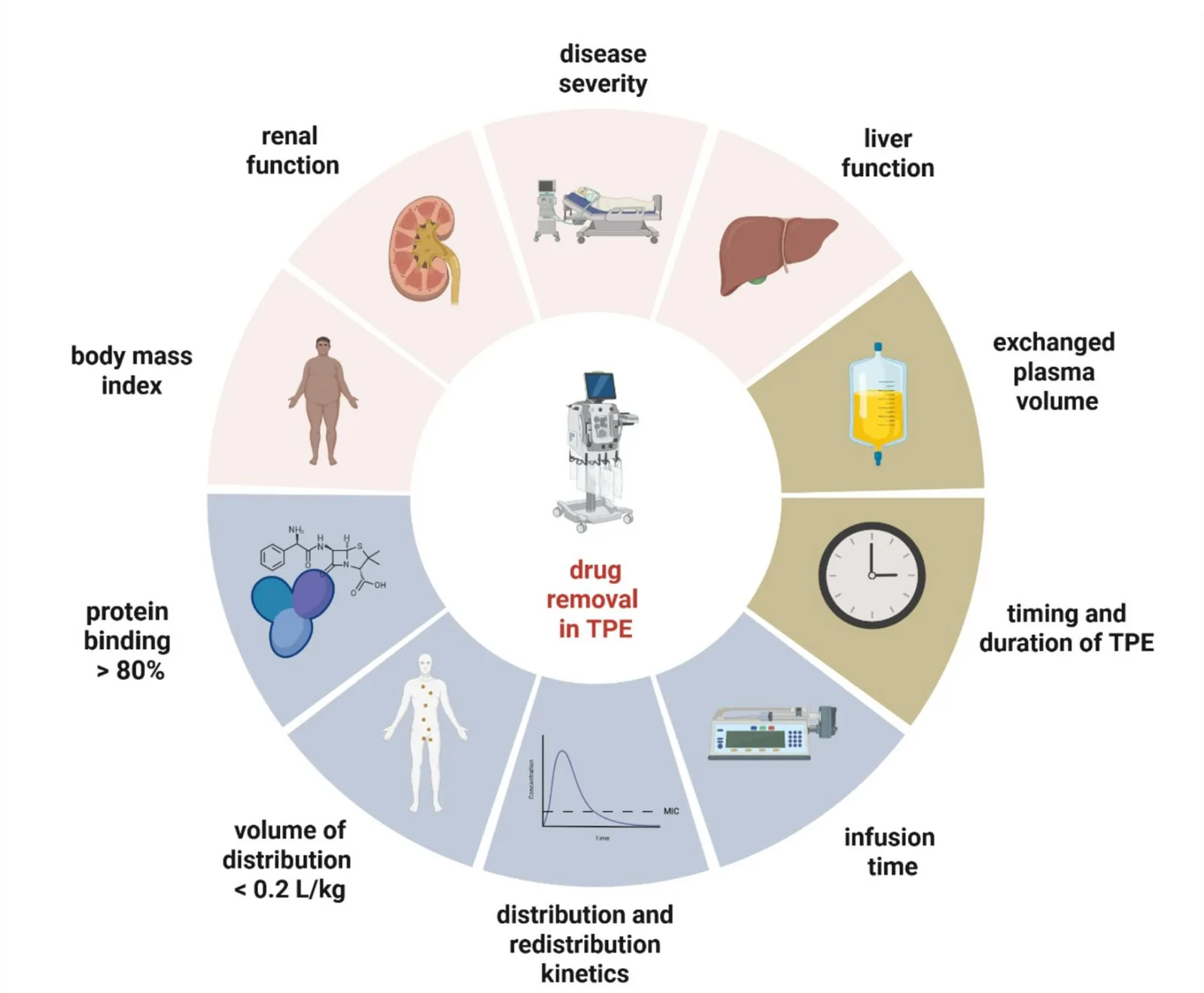
Factors influencing drug removal undergoing TPE

Figure 1 summarizes key factors influencing the impact of TPE-associated drug removal. These can be grouped into patient-specific, technical, and drug-specific factors. Patient-related variables, such as BMI, disease severity, and renal or hepatic function, are largely fixed. In contrast, technical parameters such as exchange volume, timing, and duration of TPE are modifiable and can influence the extent of drug removal. As previously discussed, scheduling TPE in relation to drug administration and adjusting its duration can alter its pharmacokinetic impact.

Among these factors, drug-specific properties such as plasma protein binding, volume of distribution, endogenous clearance, and distribution and redistribution kinetics are particularly relevant for the extent of TPE-associated drug removal. Additionally, the mode and timing of drug administration (e.g., short versus continuous infusion) in relation to TPE may influence the pharmacokinetic impact of the procedure.

Overview of patient-, drug-, and procedure-related factors influencing anti-infective drug removal undergoing TPE. Patient-related factors include disease severity, renal and liver function, and body mass index. Drug-related factors include high plasma protein binding, low volume of distribution, endogenous clearance, and distribution and redistribution kinetics. Procedure-related factors include exchanged plasma volume, timing and duration of TPE, and the timing of drug infusion in relation to the procedure. Together, these determinants influence the fraction of drug present in the intravascular compartment and therefore accessible to removal during TPE. Created in BioRender. Nachtigall, M. (2026)

### Alteration of pharmacokinetics in septic patients

Septic patients experience an imbalance of pro- and anti-inflammatory processes which leads to a rapid release of cytokines, proteases, and reactive oxygen species. These processes contribute to endothelial dysfunction, vasodilation, increased capillary permeability, impaired microcirculatory blood flow, tissue hypoxia, and eventually multi-organ dysfunction [14]. In addition to hemodynamic stabilization, early and effective anti-infective therapy is an essential part of treatment [15]. ^,^ [16]^,^ [14] Due to the dynamic nature of sepsis, drug pharmacokinetics may vary substantially between and within patients [17]. 

#### Volume of distribution

During septic shock, circulatory centralization and impaired peripheral perfusion may reduce tissue distribution and increase intravascular drug concentrations. This leads to a decrease in V_d_ and subsequently to an increase in plasma concentration. However, inflammatory processes can cause endothelial damage and capillary leakage, resulting in an increased V_d_ and comorbidities such as liver cirrhosis and heart failure may also elevate V_d_. Aggressive fluid resuscitation may increase V_d_, especially for hydrophilic drugs such as beta-lactams and aminoglycosides. Hydrophilic drugs are typically characterized by a low V_d_ and undergo primarily renal elimination. Lipophilic antimicrobials like fluoroquinolones concentrate in the intracellular space. From a TPE perspective, these opposing effects are clinically relevant because only the intravascularly accessible fraction of a drug can be directly removed during the procedure [18]. 

#### Plasma protein binding

Albumin is the most abundant plasma protein and primarily binds medium-sized, hydrophobic organic anions, including several acidic anti-infectives [19]. Albumin is synthesized in the liver. In sepsis, liver dysfunction, increased vascular permeability, redistribution of albumin into the extravascular space, and dilution by fluid resuscitation may contribute to hypoalbuminemia. As a result, the free fraction of highly albumin-bound drugs may increase. Since the unbound fraction is pharmacologically active and available for distribution, metabolism, renal elimination, and extracorporeal removal, hypoalbuminemia may alter both drug efficacy and toxicity. In contrast, α1-acid glycoprotein, a major acute-phase protein, often increases during inflammation and infection and preferentially binds neutral lipophilic and basic drugs. Thus, sepsis may shift the balance between bound and unbound drug fractions in opposite directions depending on whether albumin or α1-acid glycoprotein is the predominant binding protein [20]. ^,^ [21]

#### Lipophilicity and degree of ionization

Drug ionization and lipophilicity further influence tissue distribution and intravascular availability. Acidemia, which occurs in septic shock, affects the ionization state of weak acids and bases. Weak bases become more ionized when pH falls below their pKa, reducing their lipophilicity and potentially limiting tissue penetration. Consequently, the apparent V_d_ of basic drugs may decrease, leading to higher intravascular concentrations. Weak acids, in contrast, are often already substantially ionized under physiological conditions, and the impact of acidemia on their distribution may be less pronounced. Sepsis-associated changes in pH may therefore modify the distribution pattern of anti-infectives and alter their susceptibility to TPE-mediated removal [17, 22]. 

#### Metabolism and clearance

Endogenous drug metabolism and clearance are also frequently altered in septic patients. Hepatic drug metabolism depends on adequate liver perfusion and enzymatic capacity, both of which may be impaired during sepsis. Proinflammatory cytokines, such as TNF-alpha or IL-6, can further suppress cytochrome P450 (CYP450) enzyme activity, potentially reducing the metabolism of hepatically cleared anti-infectives [23]. Macrolides, fluoroquinolones, and azole antifungals may interact with CYP450 enzymes and thereby alter drug metabolism [24]. 

In parallel, renal function is highly variable in sepsis [25]. About 15–38% of septic patients experience acute kidney injury (AKI) [26] while most small, hydrophilic antibiotics are eliminated mainly by the kidneys [27]. Thus, AKI may reduce the clearance of small hydrophilic drugs, including beta-lactams, aminoglycosides, and glycopeptides, whereas augmented renal clearance may occur particularly in younger or hyperdynamic septic patients [28, 29]. In patients receiving RRT, extracorporeal clearance represents an additional and often substantial elimination pathway. The relative contribution of TPE to total drug clearance is therefore greatest when endogenous clearance is impaired and lowest when renal, hepatic, or extracorporeal clearance pathways already dominate drug elimination [17]. 

**Fig. 2:**
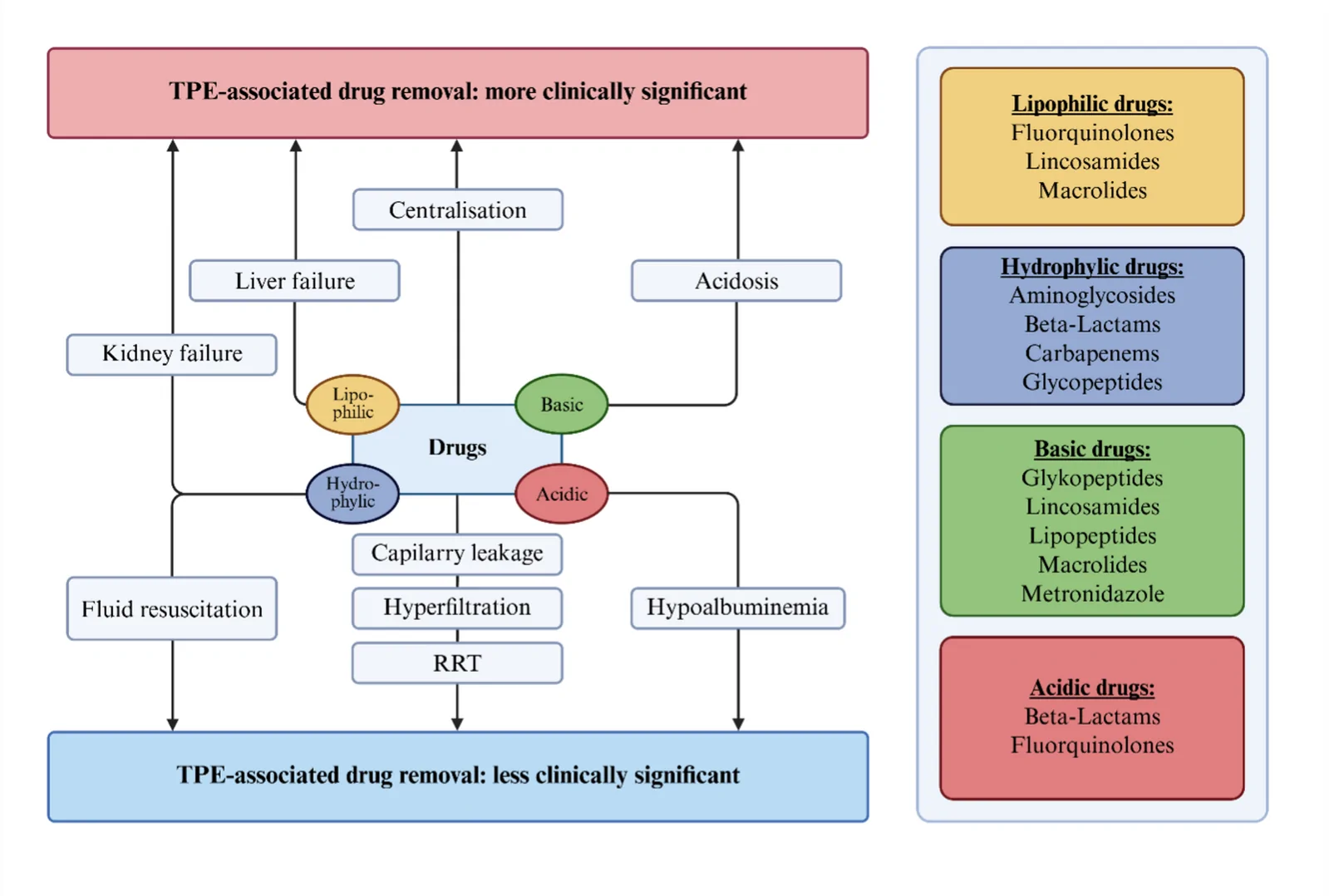
Determinants of anti-infective drug removal undergoing TPE in septic patients

It is not possible to predict the clinical relevance of TPE-associated anti-infective drug removal from a single pharmacokinetic parameter alone. The phenomenon under discussion is instead the result of the interaction between drug-specific properties (e.g. hydrophilicity, ionization, plasma protein binding, volume of distribution, and redistribution kinetics) and sepsis-associated alterations (e.g. capillary leakage, fluid resuscitation, hypoalbuminemia, organ dysfunction, acidosis, hyperfiltration, RRT, and circulatory centralization). As illustrated in Fig. 2, this theoretical framework demonstrates how these factors may shift anti-infective exposure towards either an increased or decreased clinical relevance of TPE-mediated drug removal. In this concept, hydrophilic drugs with predominant intravascular localization and limited redistribution are expected to be most susceptible to clinically relevant removal, whereas hydrophobic drugs with extensive tissue distribution are less likely to be substantially affected by TPE.

Schematic overview of drug-specific and sepsis-associated factors influencing the clinical relevance of anti-infective drug removal undergoing TPE. Drug properties such as hydrophilicity, lipophilicity, ionization, PPB, V_d_, and redistribution kinetics determine the extent to which an anti-infective is present within the intravascular compartment and therefore accessible to TPE. In septic patients, pathophysiological alterations including capillary leakage, fluid resuscitation, hypoalbuminemia, acidosis, organ dysfunction, hyperfiltration, RRT, and circulatory centralization may further modify drug distribution and clearance. The figure illustrates how these interacting factors may shift the clinical relevance of TPE-mediated drug removal toward either increased or decreased significance. This figure represents an author-derived conceptual model illustrating potential interactions between drug-specific, patient-related, and procedure-related factors and should not be interpreted as a validated causal model.

Created in BioRender. Nachtigall, M. (2026).

### Methods

#### Literature search

A structured narrative literature search was conducted to identify publications addressing anti-infective drug removal undergoing TPE. PubMed/MEDLINE, Scopus, Google Scholar, and the Cochrane Library were searched for English- and German-language literature published between January 1980 and May 2026. Eligibility criteria for publications included in the review were: Randomized controlled trials, non-randomized controlled trials, observational studies, case series and case reports. PubChem and drug monographs were additionally consulted to retrieve physicochemical and pharmacokinetic drug properties where required but were not considered primary sources for study identification.

#### Selection of literature

Articles were selected if they described TPE in humans and provided pharmacokinetic information or measured concentrations of anti-infective drugs in relation to TPE. For each included report, the following information was extracted where available: study type and sample size, anti-infective agent, exchanged plasma volume and plasma volume ratio, number of TPE sessions, timing of drug administration relative to TPE, TPE session duration, concomitant extracorporeal therapies, and reported or calculated TPE-associated drug removal. Patients of all ages and sexes were considered. No stratification was performed according to indication for TPE, comorbidities, disease severity, or concomitant therapy. These extracted study characteristics are summarized in Supplementary Table 1.

Search terms included combinations of “therapeutic plasma exchange”, “plasma exchange”, “plasmapheresis”, “apheresis”, “anti-infective”, “antibiotic”, “antimicrobial”, “pharmacokinetics”, “volume of distribution”, “half-life”, “plasma protein binding”, “drug plasma concentration”, “drug clearance”, “drug elimination”, and “drug removal”. Reference lists of relevant articles, reviews, and guidelines were screened manually to identify additional sources. Study selection was performed by two reviewers, who screened titles and abstracts followed by full-text assessment according to the eligibility criteria described above. Data extraction was performed by the same reviewers. No duplicate independent screening or data extraction was conducted.

#### Narrative synthesis and graphical presentation

**Fig. 3:**
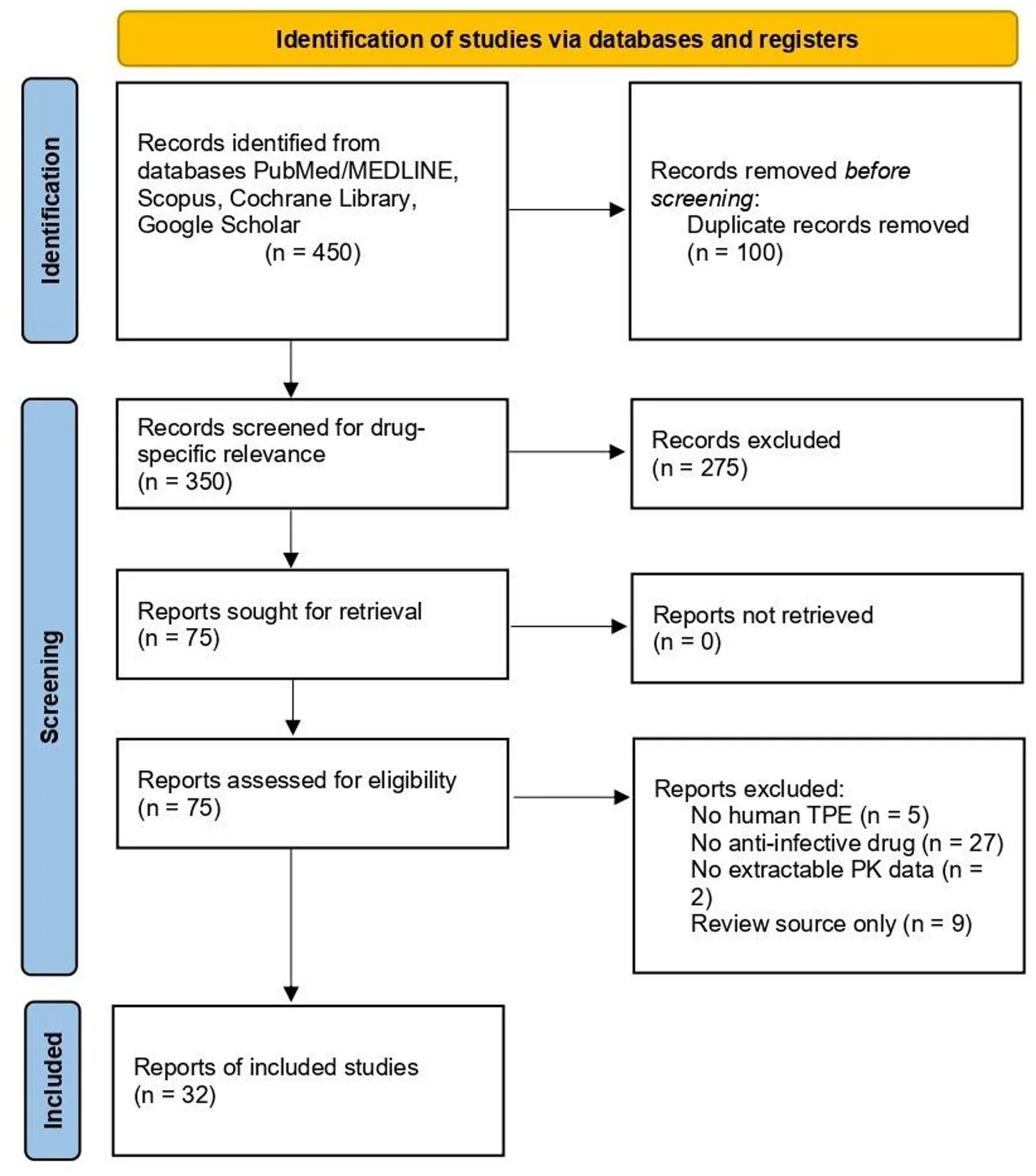
Flow diagram of the narrative review process

**Fig. 4:**
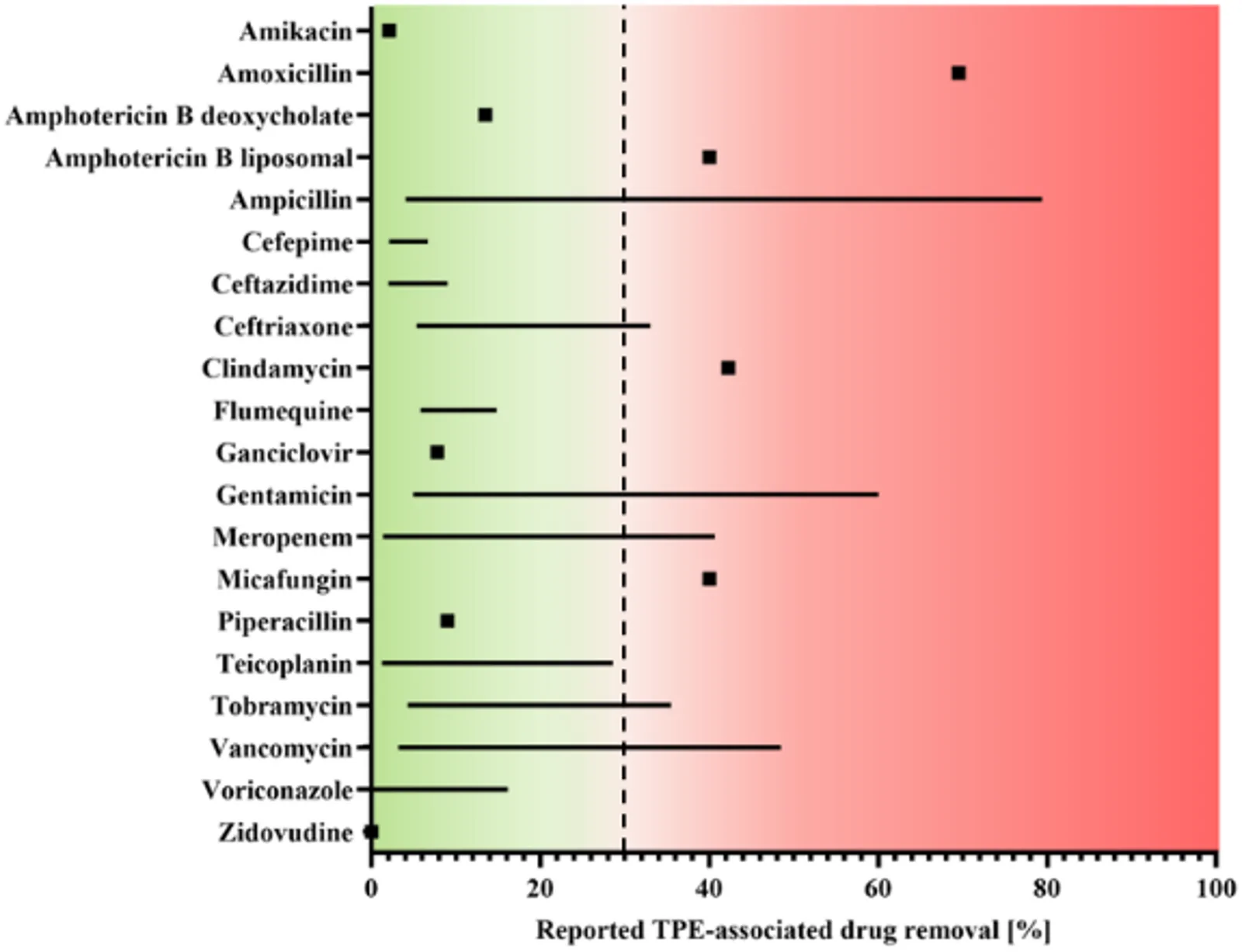
TPE-associated removal of anti-infective drugs reported in the literature

Because this work was designed as a narrative review and the available evidence consisted predominantly of heterogeneous case reports and small pharmacokinetic studies, no formal risk-of-bias tool was applied. Instead, methodological limitations were considered descriptively, including study design, sample size, completeness of pharmacokinetic and TPE reporting, timing of drug administration and sampling, and the presence of concomitant extracorporeal therapies. The literature selection process for drug-specific data extraction is presented descriptively in a flow diagram adapted from the PRISMA 2020 template (Fig. 3). Reports with extractable anti-infective concentration or pharmacokinetic data were summarized descriptively in Table 1; Fig. 4. For graphical presentation, reported or calculated percentages of drug removal undergoing TPE were displayed descriptively by individual anti-infective agent.

Flow diagram illustrating the identification and selection of literature included in this narrative review. Publications were identified through database searches and additional manual screening of reference lists, reviews, guidelines, and pharmacological sources. Articles were selected if they provided clinically or pharmacokinetically relevant information on anti-infective drug removal undergoing TPE. Adapted from the PRISMA 2020 flow diagram: Page MJ, et al. BMJ. 2021;372:n71. doi:https://doi.org/10.1136/bmj.n71. Licensed under CC BY 4.0.

## Results

A total of 450 publications were identified through the search process. After the removal of 100 duplicates, 350 records were screened for drug-specific relevance. Of these, 275 records were excluded during screening, and 75 full-text reports were assessed for eligibility. Forty-three reports were excluded because they did not involve human TPE, did not address anti-infective drugs, did not provide extractable pharmacokinetic data, or represented review sources only. Finally, 32 reports were included in the drug-specific synthesis summarized in Table 1; Fig. 4.

The included reports comprised pharmacokinetic studies and case reports describing anti-infective drug concentrations or pharmacokinetic parameters in relation to TPE. Overall, 22 anti-infective agents were investigated, with the number of patients per report ranging from one to fifteen. The available evidence was heterogeneous with respect to study design, patient population, TPE indication, procedure characteristics, and timing of drug administration. Exchanged plasma volume was reported in most studies, whereas the relative exchange volume in relation to calculated plasma volume was reported less consistently. The interval between anti-infective administration and TPE ranged from administration immediately before or after the procedure to intervals of up to 72 h. Where reported, treatment duration ranged from 54 to 480 min. Concomitant extracorporeal therapies, such as hemodialysis, were present in some reports and may have influenced drug concentrations and overall clearance. Overall, the methodological quality of the available evidence was limited by small sample sizes, heterogeneous study designs, incomplete reporting of TPE characteristics and pharmacokinetic sampling, and the frequent use of case reports. These limitations reduce the comparability of individual studies and the certainty with which TPE-associated drug removal can be interpreted.

Clinical interpretation was derived from the reported percentage of drug removal undergoing TPE, the assessment of the original authors, and the pharmacokinetic susceptibility of the respective drug. This included consideration of volume of distribution, plasma protein binding, endogenous clearance, redistribution kinetics, timing of drug administration relative to TPE, exchanged plasma volume, and concomitant extracorporeal therapies. No predefined or validated quantitative cut-offs were applied. The categories therefore represent an author-derived qualitative interpretation of the available evidence rather than a validated classification system. Based on this combined assessment, TPE-associated drug removal was classified as potentially clinically relevant in 15 reports and as likely of limited clinical relevance in 12 reports. This classification was not based on the removal percentage alone: while drugs with high reported removal, such as amoxicillin, were considered highly relevant, agents such as amphotericin B, ampicillin, clindamycin, gentamicin, and micafungin were interpreted as potentially clinically relevant because of moderate removal or favorable pharmacokinetic conditions for TPE-associated drug removal.

Overall, reported anti-infective drug removal undergoing TPE varied substantially between individual substances as shown in Fig. 4. Differing interpretations for ceftriaxone and vancomycin likely reflect differences in TPE timing, exchanged plasma volume, renal function, concomitant therapies, and drug-specific pharmacokinetic properties. Therefore, the clinical significance of TPE-associated removal should not be inferred from the percentage removed alone but interpreted together with pharmacokinetic susceptibility and procedural context. Table 1 provides a condensed clinical overview of the available evidence.

Single markers indicate individual reported values when only one extractable value was available; horizontal lines show the minimum-to-maximum range when multiple values were reported and do not represent confidence intervals. The figure is descriptive and does not represent a pooled quantitative analysis. The color gradient reflects an author-derived qualitative assessment for visual orientation only. The dashed line at 30% refers to the EXTRIP Workgroup primary criterion for classification as dialyzable (> 30% removed) and is used here as a contextual reference rather than a validated threshold for TPE-associated anti-infective drug removal [30]. Corresponding references and the underlying data are summarized in Table 1. Created in GraphPad Prism.

## Discussion

Although the use of TPE in critically ill patients is increasing, the optimal dosing of anti-infective drugs undergoing TPE remains insufficiently defined. Drug exposure in this setting is influenced by a complex interaction of procedure-related, drug-specific, and patient-specific factors. Procedure-related determinants include the exchanged plasma volume, preferably expressed in relation to the calculated plasma volume, the duration and frequency of TPE sessions, and the timing of TPE relative to drug administration. Drug-specific determinants include V_d_, PPB, endogenous clearance, and redistribution kinetics. In septic patients, additional pathophysiological alterations such as hypoalbuminemia, acidemia, capillary leakage, and organ dysfunction may further modify pharmacokinetic behavior and thereby influence the extent to which TPE affects anti-infective drug concentrations. Consequently, drug removal undergoing TPE has recently been identified as one of the most pressing research questions in the use of TPE in critically ill patients [63]. 

As shown in this review, the available evidence remains scarce, heterogeneous, and difficult to compare. Technical details of TPE are frequently incompletely reported, including exchanged plasma volume, treatment duration, timing of drug administration, and number of TPE sessions. Further, heterogeneous patient populations make it difficult to compare studies. Concurrent extracorporeal treatments, particularly RRT [64], add another layer of complexity and may substantially influence total drug clearance. Recent prospective data on vancomycin in critically ill patients receiving RRT further demonstrate that dosing requirements are influenced by actual body weight as well as RRT intensity and duration, illustrating the additional pharmacokinetic variability introduced by concomitant extracorporeal therapies [65]. These limitations restrict the comparability of published reports and make it difficult to derive reliable dosing recommendations from existing literature. Furthermore, the predominance of case reports and small pharmacokinetic studies, variability in pharmacokinetic sampling, potential publication bias, and the lack of studies linking TPE-associated drug removal to clinical outcomes further limit the certainty and generalizability of the available evidence.

Given these limitations, the following practical considerations should be understood as expert-informed interpretations based on the available pharmacokinetic evidence and physiological principles rather than as evidence-based dosing recommendations. Drugs with low volume of distribution, relevant plasma protein binding, predominant intravascular localization, or impaired endogenous clearance are more likely to be affected by TPE. However, the percentage of drug removed should not be viewed as a single parameter, as clinical relevance depends on pharmacodynamic targets, timing of TPE, redistribution kinetics, and residual renal or hepatic clearance.

For aminoglycosides, relevant TPE-associated removal has been reported in several studies. However, aminoglycosides exhibit concentration-dependent bacterial killing, and achieving an adequate peak concentration in relation to the minimum inhibitory concentration is essential for efficacy, followed by a post-antibiotic effect. Based on these pharmacokinetic considerations, administration before TPE may be considered when sufficient time is available to achieve the desired peak exposure and initial distribution. However, this represents an expert-informed strategy rather than a validated dosing recommendation, and direct evidence defining an optimal interval between aminoglycoside administration and TPE is lacking.

For beta-lactam antibiotics, the situation differs because their efficacy is primarily related to the time during which free drug concentrations remain above the minimum inhibitory concentration. Therefore, TPE-associated removal may be clinically relevant if it reduces concentrations during the dosing interval, particularly in patients with unstable pharmacokinetics, increased V_d_, or concomitant organ dysfunction. In such cases, supplemental dosing after TPE may be considered on an individual basis, particularly when substantial drug removal is expected or has been reported; however, direct evidence supporting routine supplemental dosing is limited. Overall, timing of TPE relative to anti-infective administration is a central determinant of clinical relevance and should be systematically documented in future studies.

In clinical practice, however, such timing considerations must be integrated into a time-critical ICU workflow. In newly admitted patients with severe sepsis or septic shock, blood culture collection, lactate measurement, and early antibiotic administration are prioritized within the first hours of care [66]. Blood type testing usually requires 30 min [67] and thawing time for FFPs is about 15–30 min [68]. The preparation time of a TPE machine, which includes setup and priming ranges between 11 and 23 min [11]. In some clinical settings, the preparation required before initiation of TPE may result in a time interval between the first anti-infective dose and the start of TPE, thereby allowing initial drug distribution to occur. However, this interval is institution- and situation-dependent and should not be assumed to provide reliable protection from TPE-associated drug removal.

Therapeutic drug monitoring (TDM) may support individualized dosing but should be interpreted cautiously. Plasma concentrations do not necessarily reflect drug exposure at the site of infection, particularly in septic patients with altered perfusion, capillary leakage, organ dysfunction, or concomitant extracorporeal therapies. Thus, TDM should be considered one component of individualized dosing rather than a direct surrogate for target-site exposure [69]. TDM may be particularly useful for beta-lactams, vancomycin, and selected antifungal agents in critically ill patients, in whom pharmacokinetic variability and TPE-associated drug removal may complicate standard dosing. Where available, drug concentrations obtained in relation to the timing of TPE may support individualized dose adjustment and interpretation of procedure-associated changes in exposure.

## Conclusion

Anti-infective therapy is a cornerstone of sepsis treatment and must be administered early and effectively. TPE may alter plasma concentrations of anti-infective drugs, particularly when drugs have a low volume of distribution, relevant plasma protein binding, predominant intravascular distribution, or impaired endogenous clearance. However, the clinical relevance of drug removal undergoing TPE cannot be predicted from the percentage removed alone. It must be interpreted in relation to pharmacodynamic targets, timing of drug administration, exchanged plasma volume, redistribution kinetics, organ function, and concomitant extracorporeal therapies.

Whenever clinically feasible, the timing of TPE and anti-infective administration may be coordinated to reduce the potential for TPE-associated drug removal during early distribution. For aminoglycosides, administration before TPE may be considered if sufficient time is available to achieve adequate peak exposure and initial distribution. For beta-lactams, supplemental dosing after TPE may be considered on an individual basis when substantial drug removal is expected or has been reported. These strategies should be regarded as expert-informed considerations rather than evidence-based dosing recommendations.

Preparation for TPE may create an interval between initial anti-infective administration and initiation of the procedure; however, the duration of this interval varies across institutions and clinical situations, and its effect on TPE-associated drug removal cannot be generalized. Future studies should prospectively document TPE parameters, dosing times, sampling times, organ function, and concomitant extracorporeal therapies to support more reliable dosing recommendations.

**Table 1:** Condensed clinical overview of reported anti-infective drug removal undergoing TPE

Anti-infective	*n*	Reported drug removal [%]	Author-derived clinical interpretation	Data basis
Acyclovir [31]	1	NR	Significant	Case report
Amikacin [32]	1	2.09	NS	Case report
Amoxicillin [33]	1	69.52	Significant	Case report
Amphotericin B deoxycholate [34]	1	13.5	NS	Case report
Amphotericin B deoxycholate [35]	1	NR	NS	Case report
Amphotericin B liposomal [36]	1	40	Significant	Case report
Ampicillin [37]	15	4.05–79.4	Moderate	PK study
Cefepime [38]	9	2.1–6.7	NS	PK study
Ceftazidime [39]	11	2–9	NS	PK study
Ceftriaxone [40]	1	5.4	NS	Case report
Ceftriaxone [41]	12	8.2–33	Moderate	PK study
Chloramphenicol [42]	1	NR	NR	Case report
Clindamycin [33]	1	42.25	Significant	Case report
Flumequine [43]	8	5.8–14.8	NS	PK study
Ganciclovir [44]	1	7.8	NS	Case report
Gentamicin [45]	7	18.5	Moderate	PK study
Gentamicin [46]	12	25.7	Moderate	PK study
Gentamicin [47]	7	4.95	NS	PK study
Gentamicin [37]	15	16.6–60	Moderate	PK study
Meropenem [32]	2	1.37–2.16	NS	Case report
Meropenem [48]	11	4.15–40.66	Moderate	PK study
Micafungin [49]	1	40	Significant	Case report
Piperacillin [50]	1	9	NS	Case report
Teicoplanin [51]	12	11.1–28.6	Moderate	PK study
Teicoplanin [32]	1	1.25–5.4	NS	Case report
Tobramycin [52]	1	6.8–10.8	NS	Case report
Tobramycin [53]	1	35.5	Moderate	Case report
Tobramycin [54]	2	4.3–6.1	NS	PK study
Vancomycin [55]	1	27	Moderate	Case report
Vancomycin [56]	12	3.2–9.5	NS	PK study
Vancomycin [57]	1	27	Moderate	Case report
Vancomycin [58]	1	NR	Moderate	Case report
Vancomycin [59]	1	48.5	Significant	Case report
Voriconazole [32]	1	9.26–16.15	Moderate	Case report
Voriconazole [60]	1	NR	NS	Case report
Voriconazole [61]	1	0.02	NS	Case report
Zidovudine [62]	1	0.01	NS	PK study / case report

This table summarizes reported anti-infective drug removal undergoing TPE. Reported drug removal is presented as a single value or range when multiple observations were available. The clinical interpretation reflects the reported extent of removal together with the assessment provided by the original authors where available. The terms “significant”, “moderate”, and “insignificant” represent author-derived qualitative assessments of potential clinical relevance and do not refer to statistical significance or validated quantitative thresholds. The table is intended as a descriptive overview rather than a pooled quantitative analysis

Abbreviations: PK, pharmacokinetic; NR, not reported; NS, not significant; n, number of patients

## Supplementary Information

Below is the link to the electronic supplementary material.

Supplementary Material 1

## Data Availability

No datasets were generated or analysed during the current study.
