# Supplementary material for: Dosing of anti-infective drugs in patients undergoing therapeutic plasma exchange – considerations for practical application: a narrative review
Source: Crit Care. 2026 Sep 24;30:493. doi: 10.1186/s13054-026-06306-0 (PMC13613798; doi:10.1186/s13054-026-06306-0)
Supplement: Supplementary file 1 [file 13054_2026_6306_MOESM1_ESM.docx]

**Supplementary Table 1. Technical characteristics of included reports on anti-infective drug removal during therapeutic plasma exchange**

| Reference | Anti-infective | n | Study type | Exchanged plasma volume [mL] | Plasma volume ratio | Number of TPE | TPE-drug interval | Duration of TPE [min] | Reported drug removal [%] | Additional extracorporeal therapy |
| --- | --- | --- | --- | --- | --- | --- | --- | --- | --- | --- |
| [31] | Acyclovir | 1 | Case report | 50 mL/kg | NR | 5 | 6 h | 120 | NR | NR |
| [32] | Amikacin | 1 | Case report | 3315 | NR | 1 | 21.6 h | 90 | 2.09 | NR |
| [33] | Amoxicillin | 1 | Case report | 2200 | NR | 3 | during continuous infusion | NR | 69.52 | ECMO; CVVHDF |
| [34] | Amphotericin B deoxycholate | 1 | Case report | NR | NR | 4 | 72 h | NR | 13.5 | NR |
| [35] | Amphotericin B deoxycholate | 1 | Case report | NR | NR | 4 | 36 h | NR | NR | NR |
| [36] | Amphotericin B liposomal | 1 | Case report | NR | NR | 1 | 38 h | 120 | 40 | Hemodialysis |
| [37] | Ampicillin | 15 | PK study | 115–450 | 2 | 1 per observation | 2–10 h | 40–105 | 4.05–79.4 | NR |
| [38] | Cefepime | 9 | PK study | 2500–3500 | NR | 1 | 1.5 h | 94–209 | 2.1–6.7 | NR |
| [39] | Ceftazidime | 11 | PK study | 2050–2150 | NR | 1 | 15–120 min | 60–110 | 2–9 | NR |
| [40] | Ceftriaxone | 1 | Case report | 3000 | NR | 3 | 12 h | 150 | 5.4 | NR |
| [41] | Ceftriaxone | 12 | PK study | 2800–4300 | NR | 1 per PK observation | 0 or 6 h | 65–150 | 8.2–33 | NR |
| [42] | Chloramphenicol | 1 | Case report | 5400 total | NR | multiple | 29 h | NR | NR | NR |
| [33] | Clindamycin | 1 | Case report | 2200 | NR | 3 | during continuous infusion | NR | 42.25 | ECMO; CVVHDF |
| [43] | Flumequine | 8 | PK study | 2500–4100 | 1–1.5 | 1 | 20 h | ~120 | 5.8–14.8 | NR |
| [44] | Ganciclovir | 1 | Case report | 3555 | NR | 2 analyzed | NR | NR | 7.8 | CVVHDF |
| [45] | Gentamicin | 7 | PK study | NR | 2 | 8 | 9.5 h | 54.3 | 18.5 | NR |
| [46] | Gentamicin | 12 | PK study | NR | 2 | 1 | NR | 55 | 25.7 | NR |
| [47] | Gentamicin | 7 | PK study | 90 mL/kg × 2 | 2 | 7 | 7 h | 56 | 4.95 | NR |
| [37] | Gentamicin | 15 | PK study | 115–450 | 2 | 1 per observation | 2–10 h | 40–105 | 16.6–60 | NR |
| [32] | Meropenem | 2 | Case report | 3381–4455 | NR | 1 per observation | 2.8–5.3 h | 90–130 | 1.37–2.16 | NR |
| [48] | Meropenem | 11 | PK study | 2408–4874 | NR | 1 | 0 (at TPE start) | 105–191 | 4.15–40.66 | NR |
| [49] | Micafungin | 1 | Case report | 3200 | 1 | 4 | 2.5 h | 480 | 40 | NR |
| [50] | Piperacillin | 1 | Case report | 2479 | NR | 2 | 0 (continuous infusion) | 81.5 | 9 | NR |
| [51] | Teicoplanin | 12 | PK study | 2660–5486 | NR | 1 per patient | 0 | NR | 11.1–28.6 | NR |
| [32] | Teicoplanin | 1 | Case report | 4293–4455 | NR | 1 per observation | 17.2–25.8 h | 113–130 | 1.25–5.4 | NR |
| [52] | Tobramycin | 1 | Case report | 1700–2170 | NR | 2 | 1 h; 18 h | NR | 6.8–10.8 | Hemodialysis |
| [53] | Tobramycin | 1 | Case report | 5850 | NR | 1 | 6 h | 138 | 35.5 | NR |
| [54] | Tobramycin | 2 | PK study | 1565–2057 | NR | 3 total | NR | NR | 4.3–6.1 | NR |
| [55] | Vancomycin | 1 | Case report | NR | NR | 2 | NR | NR | 27 | Hemodialysis |
| [56] | Vancomycin | 12 | PK study | 2719–5704 | 1 | 1 per patient | 0–134.25 h | 80–180 | 3.2–9.5 | NR |
| [57] | Vancomycin | 1 | Case report | NR | NR | 5 | NR | NR | 27 | NR |
| [58] | Vancomycin | 1 | Case report | 3487 | NR | 1 | 24 h | 120 | NR | NR |
| [59] | Vancomycin | 1 | Case report | 3100 | 1 | 14 | NR | NR | 48.5 | NR |
| [32] | Voriconazole | 1 | Case report | 4293–4455 | NR | 1 per observation | 0.3–7.8 h | 113–130 | 9.26–16.15 | NR |
| [60] | Voriconazole | 1 | Case report | 3445 | NR | 13 | 2.5 h | 115 | NR | NR |
| [61] | Voriconazole | 1 | Case report | 3300–3600 | NR | 1 | 5 min | 120–150 | 0.02 | NR |
| [62] | Zidovudine | 1 | PK study / case report | 2913 | NR | 3 | 4 h | 60 | 0.01 | NR |

*Abbreviations*: TPE, therapeutic plasma exchange; PK, pharmacokinetic; NR, not reported; ECMO, extracorporeal membrane oxygenation; CVVHDF, continuous veno-venous hemodiafiltration.

Each row represents one source/anti-infective combination. Ranges indicate the lowest and highest values extracted from the respective report.
